# Nogo-A antibody delivery through the olfactory mucosa mitigates experimental autoimmune encephalomyelitis in the mouse CNS

**DOI:** 10.1038/s41420-023-01588-7

**Published:** 2023-08-09

**Authors:** Vincent Pernet, Sandrine Joly, Sebastian Spiegel, Ivo Meli, Sherif Idriss, Frank Maigler, Julius Baya Mdzomba, Anna K. Roenneke, Alessandra Franceschini, Ludovico Silvestri, Francesco S. Pavone, Martino Calamai, Katharina Schindowski, Andrew Chan

**Affiliations:** 1 Department of Neurology, Inselspital, Bern University Hospital, University of Bern, Bern, Switzerland; 2 Center for experimental neurology (ZEN), Bern University Hospital, University of Bern, Bern, Switzerland; 3 Centre de recherche du CHU de Québec-Université Laval and Department of Molecular Medicine, Faculté de médecine, Université Laval, Québec, Québec, Canada; 4 Department of Ophthalmology, Inselspital, Bern University Hospital, University of Bern, Bern, Switzerland; 5 Institute of Applied Biotechnology, Biberach University of Applied Science, Hubertus-Liebrecht-Strasse 35, Biberach, Germany; 6 Department of Biomedical Research, University of Bern, Bern, Switzerland; 7 Graduate School for Cellular and Biomedical Sciences, University of Bern, Bern, Switzerland; 8 LENS- European Laboratory for Non-Linear Spectroscopy, University of Florence, Sesto-Fiorentino (Firenze), Italy; 9 National Institute of Optics - National Research Council (CNR-INO), Sesto Fiorentino, Italy

**Keywords:** Drug delivery, Multiple sclerosis

## Abstract

Systemic administration of Nogo-A-neutralizing antibody ameliorates experimental autoimmune encephalomyelitis (EAE), an animal model of multiple sclerosis. However, the blood-brain barrier (BBB) is a major obstacle limiting the passage of systemically applied antibody to the CNS. To bypass the BBB, in the present study we tested the intranasal route of administration by targeting the olfactory mucosa with the Nogo-A-blocking antibody 11C7 mAb in myelin oligodendrocyte glycoprotein-induced EAE. Antibodies were specifically administered onto the olfactory mucosa using a microcatheter. Antibody distribution was examined in the CNS by ELISA and light-sheet microscopy. The effects of 11C7 mAb on Nogo-A signaling were assessed by Western blotting. EAE-induced deficits were monitored daily. Demyelination was observed on spinal cord histological sections. Gene expression changes were followed by trancriptomic analyses. A sensitive capture ELISA revealed a rapid and widespread distribution of 11C7 mAb in the CNS, including the olfactory bulb, the cerebellum and the lumbar spinal cord, but not in the CSF. Light-sheet microscopy allowed to observe antibody accumulation in the parenchyma, thus demonstrating nose-to-brain transfer of IgG. At the functional level, the widespread penetration of 11C7 mAb in the CNS, including the thoracolumbar spinal cord, resulted in the improvement of motor symptoms and in the preservation of myelin in the spinal cord of EAE mice. This was accompanied by Nogo-A signaling downregulation, as reflected by the decreased level of phosphorylated cofilin observed by Western blotting in the cerebellum. In the brain of EAE score-matched animals, 11C7 modified the expression of genes that can influence neurotransmission and cognitive functions, independently of the demyelination phenotype in the spinal cord. In conclusion, our data show the feasibility of olfactory mucosa-directed administration for the delivery of therapeutic antibodies targeting CNS antigens in EAE mice.

## Introduction

Current disease modifying therapies in multiple sclerosis allow to attenuate the immune response and to slow down clinical disease progression [1, 2]. However, already accrued neuronal and myelin damage of multiple sclerosis remain untreatable. Therapies that stimulate neuronal and myelin repair and thus preserve or restore major neurological functions in progressive multiple sclerosis are yet to be established [3].

Nogo-A and its receptors are promising molecular targets for the treatment of neurodegenerative diseases [4]. Originally, Nogo-A has been described as a potent myelin-associated inhibitor of neuronal plasticity in the CNS [4, 5]. After spinal cord injury, its neutralization with function-blocking antibodies, such as the 11C7 mAb, can promote axonal outgrowth and locomotor recovery in rodents [6, 7]. In addition, studies showed that targeting Nogo-A or its receptors with antibodies, siRNA or pharmacological blockers induces neurological recovery in experimental models of multiple sclerosis [8–11]. For example, intravenous bolus injections of a blocking antibody binding the delta 20 domain of Nogo-A [12], similarly to 11C7 mAb, dramatically reduced the severity of experimental autoimmune encephalomyelitis (EAE) [10]. In this last study, the beneficial effects disappeared in the chronic phase, most likely because of antibody clearance from brain and spinal cord tissues. To maintain therapeutic effects, a sustained delivery of antibody and its widespread distribution may be required in the CNS. However, with conventional routes of administration, such as intravenous injections, only a small fraction of 11C7 IgG (0.007–0.05%) can enter the rat CNS [13]. Although more efficient, the intrathecal delivery is invasive and presents potential complications such as cerebrospinal fluid (CSF) leakage and infections [14]. The establishment of non-invasive administration methods for IgGs targeting CNS antigens, such as Nogo-A, is thus a major challenge for the treatment of chronic neurodegenerative diseases.

The intranasal administration route allows a variety of large biologics [15], including IgG [16, 17], to reach the brain where they can exert beneficial effects in animal models of stroke [18] and Alzheimer’s disease [19, 20]. The intranasal pathway has been shown to be effective for CNS delivery of interferon β [21], nerve growth factor (NGF) [19], insulin-like growth factor-1 (IGF-I) [18], and anti-amyloid β (Aβ) scFv antibody [20]. Upon intranasal delivery, full IgG could be detected at therapeutically relevant concentrations in remote brain areas, such as the brainstem [16]. However, as the nasal epithelia are not uniform, drugs administered in the nasal cavity can follow different routes of transport depending on their absorption in the respiratory or the olfactory regions. Current approaches of intranasal drug administration are not region-specific but rather lead to flooding of the nostrils. We have recently demonstrated that a region-specific microcatheter-based delivery to the olfactory mucosa enhances brain penetration without systemic (peripheral) effects, in contrast to respiratory mucosa-directed delivery [17]. Here, we set out to investigate if such nose-to-brain antibody delivery can functionally impact an experimental model of autoimmune inflammation characterized by predominant spinal cord pathology.

## Results

### Intranasal administration on the olfactory mucosa allows widespread and rapid IgG transfer to the mouse CNS

**Fig. 1:**
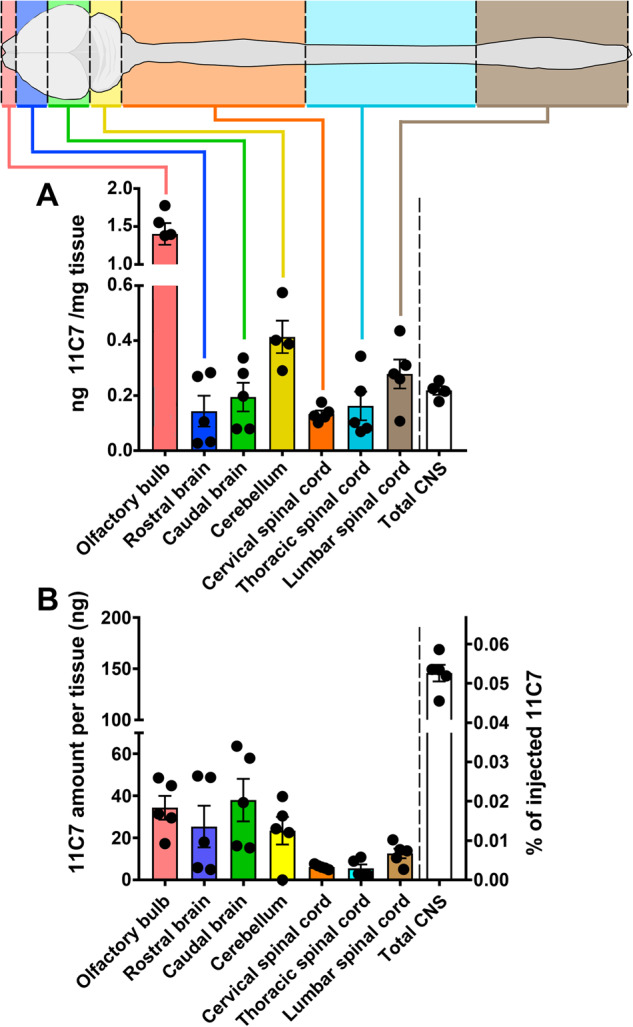
Detection of 11C7 by capture ELISA in the mouse CNS. 11C7 was detected by capture ELISA, using human Nogo-A delta 20 fragment as a bait, in the brain and in the spinal cord of naive mice after 5 consecutive days of intranasal administration. **A** The concentration of 11C7 was the highest in the olfactory bulb, the cerebellum and in the lumbar spinal cord. **B** When expressed per tissue, brain structures contain the highest amounts of 11C7. Values are mean ± S.E.M. Four-five mice have been used for each measurement.

**Fig. 2:**
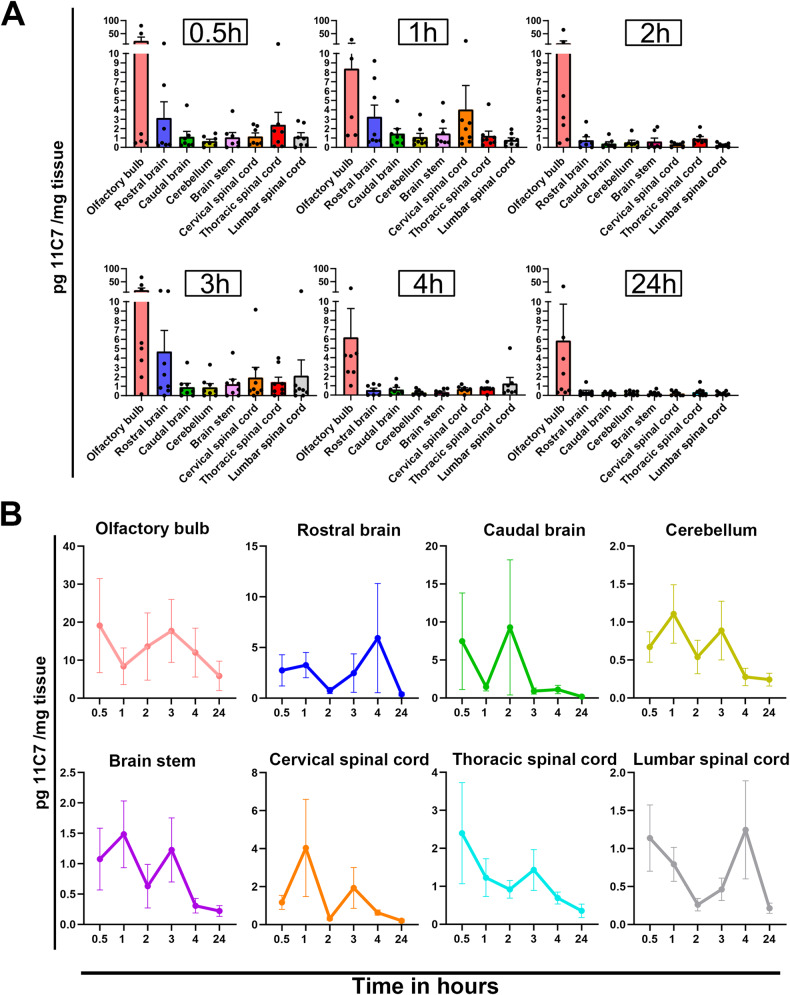
11C7 pharmacokinetic in the mouse CNS. After single intranasal administrations, time-dependent 11C7 concentration changes were followed in the mouse CNS by capture ELISA using rat Nogo-A delta 20 fragment as a bait. **A** The level of 11C7 quickly increased in rostral CNS structures close to the site of administration, such as the olfactory bulb. In addition, 11C7 was detectable in spinal cord segments as early as 0.5 h after olfactory mucosa-targeted application, suggesting fast and widespread CNS distribution of the antibody using this route of administration. **B** 11C7 kinetic analysis suggests a decline starting 3 or 4 h post administration and confirmed at 24 h.

**Fig. 3:**
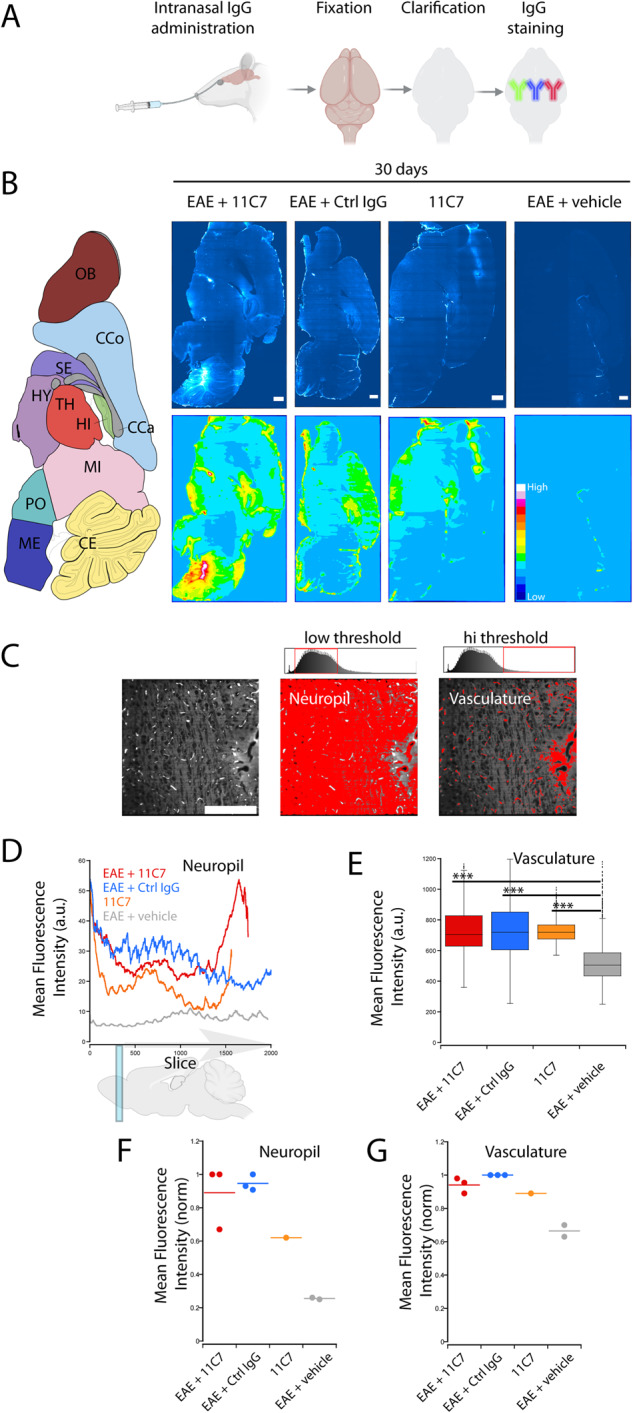
Brain distribution of IgG after intranasal administration. **A** Methodological approach for whole brain processing with CLARITY after the administration of IgG on the olfactory mucosa (cartoon produced with the Biorender software). **B** Representative images of IgG immunostaining on sagittal optical sections of mouse brains acquired by light-sheet microscopy, after tissue clearing and IgG detection by immunofluorescence. Scale bar = 1 mm. **C** Two different thresholds were applied to discriminate IgGs accumulated in the neuropil (low fluorescence intensity) and the vasculature (high fluorescence intensity). Scale bar = 500 μm. **D** Neuropil profiles of IgG fluorescence between the olfactory bulb and the cerebellum (1 brain/condition). **E** Average IgG fluorescence in blood vessels (1 brain/condition). Student’s *t*-test, ***P < 0.001. **F, G** Average fluorescence intensity of the neuropil (**F**) and vasculature (**G**) of brains undergoing different treatments. Each dot corresponds to the mean fluorescence intensity of a brain from independent experiments, normalized to the highest mean value for each set of experiments. *n* = 3 brains for EAE + 11C7, 3 for EAE + Ctrl IgG, 2 for EAE + vehicle, 1 for 11C7.

A refined microcatheter-based delivery method allowed targeted antibody application on the olfactory mucosa [17]. With this method, we examined by capture ELISA the distribution of 11C7 in the plasma, and in the CNS of naive mice treated for 5 consecutive days with 60 µg/day of control IgG or 11C7 (Fig. 1). This assay was developed by immobilizing recombinant human delta 20 fragment, the Nogo-A region recognized by 11C7 [6, 7], in 96-well plates (Fig. 1). In the plasma, the concentration of 11C7 was 140.54 ± 18.31 µg/mL (mean ± S.E.M.; *n* = 5). Interestingly, the plasma of mice treated with control IgG contained 49.9 ± 3.27 µg/mL of endogenous antibodies binding delta 20 (*n* = 3), suggesting that anti-Nogo-A antibodies are naturally produced in mice at relatively low level, as recently reported by another group [22]. In the CSF, 11C7 was undetectable (Table S1). In brain tissues, 11C7 was detected in CNS areas distant from the site of injection, not only in the olfactory bulb, but also in the cerebellum and the lumbar spinal cord (Fig. 1A). When expressed in total amount per CNS tissues, 11C7 was more abundant in brain structures whose size was much bigger than that of spinal cord segments (Fig. 1B). The time-course of 11C7 distribution was established within the first 24 h following single intranasal delivery of 60 μg of antibody (Fig. 2A). For this, capture ELISA measurements were carried out using rat Nogo-A aa 623-640 fragment as a bait, corresponding to 11C7 epitope [12], that allowed specific binding of 11C7 but yielded lower values than the use of the human delta 20 fragment (Fig. 1). Remarkably, 11C7 was detected in CNS tissues, including spinal cord segments, as early as 0.5 h after its application on the olfactory mucosa (Fig. 2A), suggesting fast and widespread antibody distribution in the CNS with this route of administration. 11C7 was strongly decreased in all tissues at 24 h, except in the olfactory bulb (Fig. 2B). Next, in order to determine if antibodies reach the brain parenchyma in intact and EAE brains after 30 days of treatment, light-sheet microscopy was carried out on cleared tissues [23]. This approach allowed to observe a bright signal for 11C7 and control IgG compared with vehicle administration in EAE mice (Fig. 3B, D–G). The levels of antibodies were separately evaluated in the parenchyma and in the vasculature (Fig. 3C–G). Blood vessels exhibited higher intensities than the neuropil (Fig. 3C–E). In the neuropil, the mean fluorescence intensity of IgGs, measured on coronal optical sections, appeared stronger in the olfactory bulb, the cerebellum and at the level of the striatum, in agreement with ELISA measurements in naive mice (Fig. 3D). In blood vessels, the increased fluorescent signal observed after 11C7 and control IgG administration suggests efflux of antibodies in the circulation (Fig. 3E, G). Together, capture ELISA and light-sheet microscopy results indicate that the intranasal, olfactory mucosa-directed administration is an efficacious mode of delivery, ensuring widespread and rapid distribution of IgGs in the brain and in the spinal cord of naive and EAE-diseased mice, without clear involvement of CSF transport.

### Intranasal administration of Nogo-A-blocking antibody attenuates EAE

**Fig. 4:**
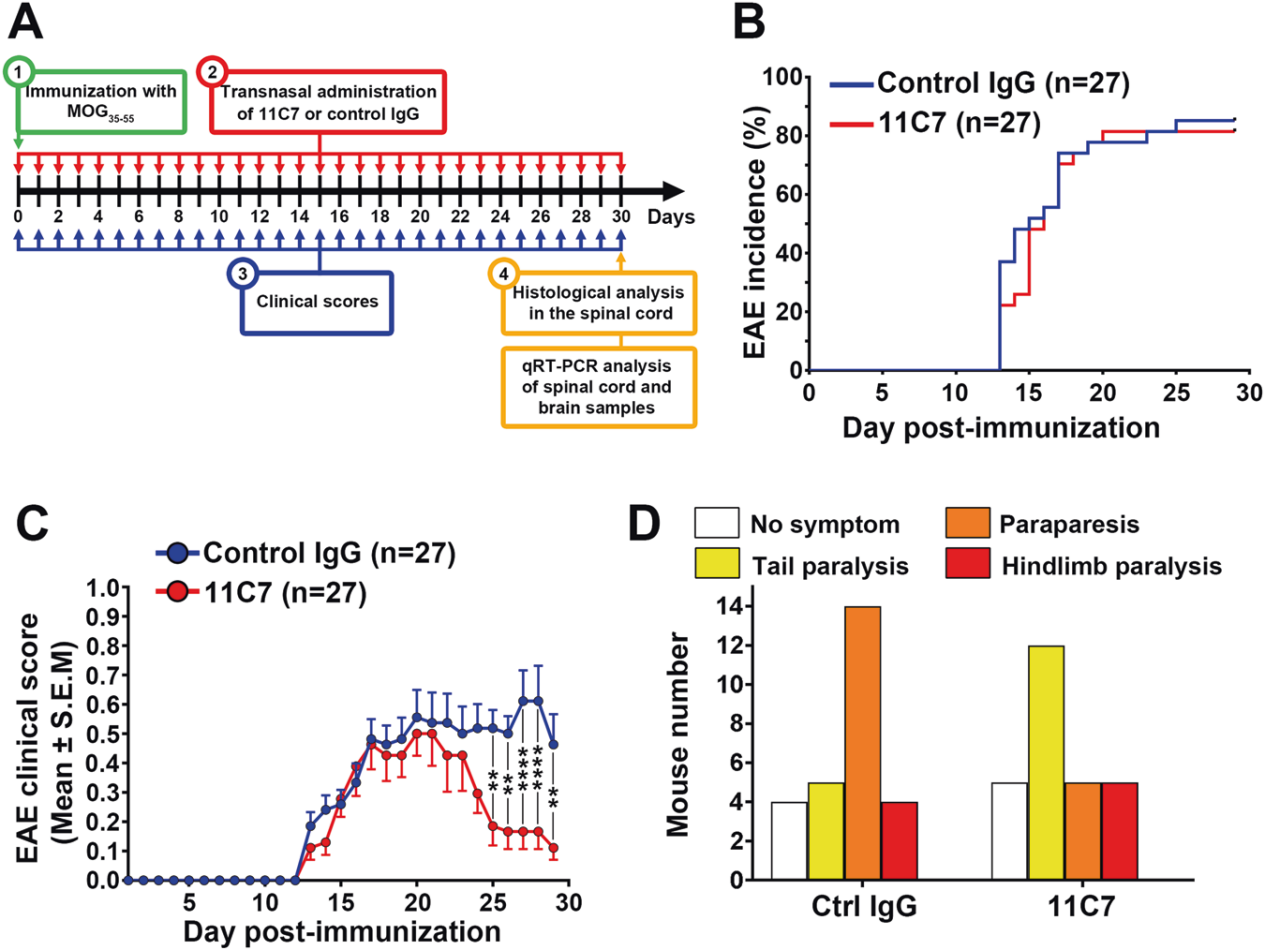
Intranasal administration of 11C7 attenuates the motor symptoms of experimental autoimmune encephalomyelitis (EAE). **A** Experimental timeline. **B** The incidence of EAE clinical scores did not differ between the two treatments. **C** However, 11C7 markedly reduced the level of clinical scores 24–30 days after EAE induction compared with control IgG treatment. **D** The analysis of EAE score distribution revealed a lower number of mice with paraparesis in the 11C7 group. Statistics. C Two-way ANOVA, Bonferroni’s multiple comparison test, **P < 0.01, ***P < 0.001, ****P < 0.0001. D Mann–Whitney U-test.

EAE clinical signs were monitored in C57BL/6J mice receiving intranasal antibody administrations for 30 consecutive days (Fig. 4A). In this experimental disease paradigm, symptoms appear first in the tail and progressively affect the hindlimbs and forelimbs due to caudal-to-rostral spinal cord demyelination [24]. Our data showed that the EAE incidence and day of onset were not influenced by 11C7 (~81% incidence out of 27 mice) relative to control IgG (~85% incidence out of 27 mice) (Fig. 4B), suggesting that anti-Nogo-A antibody does not prevent EAE induction after MOG immunization. In contrast, the administration of 11C7 strongly decreased the level of EAE clinical scores in the chronic phase when compared with control treatment (Fig. 4C). Motor function improvement started 25 days after MOG injection (Fig. 4C). The examination of the animal distribution according to EAE clinical scores, 30 days after MOG immunization, revealed that the beneficial effects of 11C7 come mainly from a reduction in the number of mice with paraparesis (Fig. 4D). Based on these data, we conclude that the intranasal pathway is an efficient route of administration for 11C7, allowing to mitigate the functional signs of EAE attributable to thoracolumbar spinal cord pathology.

### Myelin preservation correlates with functional recovery

**Fig. 5:**
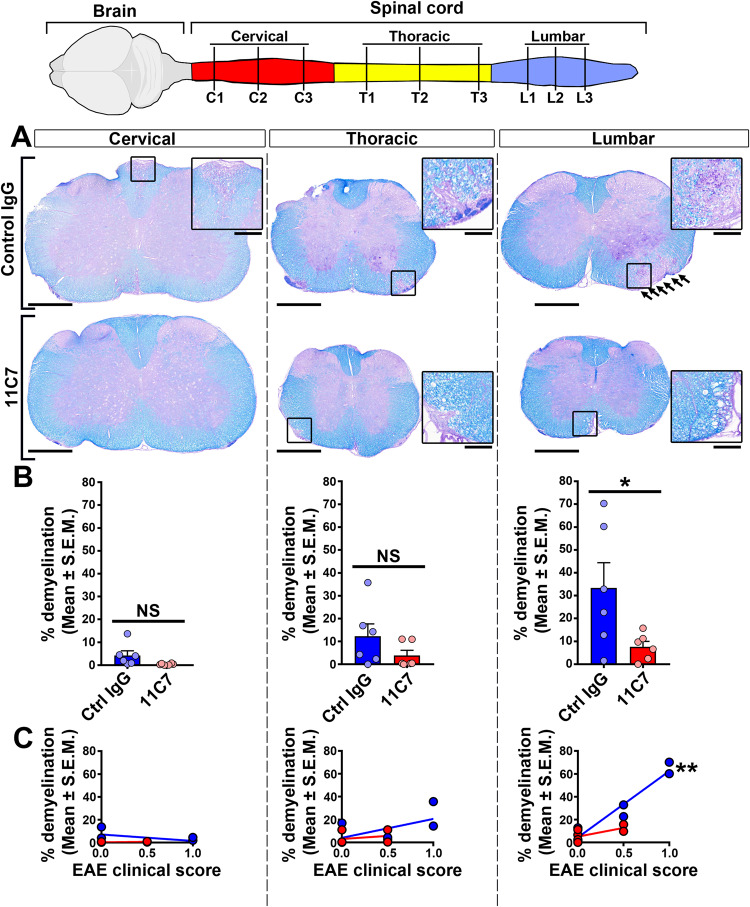
Demyelination is reduced in the spinal cord of mice treated with 11C7. Myelin was examined in the cervical, thoracic and lumbar segments on transverse sections of spinal cords stained with LFB-PAS, 30 days after MOG-induced EAE. **A** LFB-PAS stainings revealed a predominant demyelination in the lumbar spinal cord of mice treated with control IgG. In contrast, LFB-free areas were smaller in mice treated with 11C7. **B** Quantitatively, the % of demyelination was statistically lower in the lumbar spinal cord of mice receiving 11C7 than in control littermates (*P < 0.05; NS not significant). **C** The % of demyelination in lumbar spinal cord correlated with the EAE clinical scores of control IgG-injected mice (Pearson *r* = 0.9635; **P < 0.01). For quantitative analysis, three sections were used per animal in each segment, as shown in the top cartoon. Scale bars: A = 500 μm, close-ups= 200 μm.

**Fig. 6:**
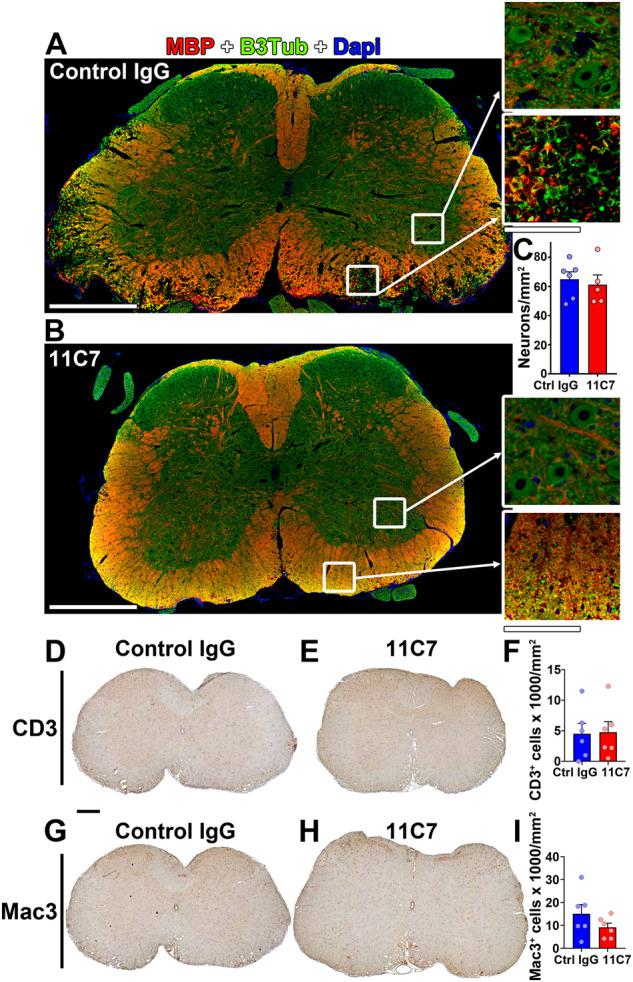
Neuronal survival and immune cell infiltrations are not influenced by 11C7. **A, B** Spinal cord sections were immunofluorescently stained for myelin basic protein (MBP) and beta 3 tubulin (B3Tub) to visualize myelin and neuronal structures in the white and gray matters respectively. Extended myelin damage was observed in the white matter of EAE mice treated with control IgG (**A**), although 11C7 treatment was associated with modest myelin damage (**B**). **C** The density of large B3Tub-positive neurons was quantified to determine if 11C7 protected motoneurons in the ventral gray matter. Quantitatively, the number of neurons did not differ between the 2 experimental groups. **D**–**I** T cell and monocyte infiltration was observed in the lumbar spinal cord of EAE mice using CD3 and Mac3 as specific markers, respectively. The density of CD3^+^ (**F**) and Mac3^+^ (**I**) cells did not differ between control and 11C7 treatments. Each dot represents a different animal. Scale bars: A, B = 500 μm, close-ups= 100 μm; D, E, G, H = 200 μm.

**Fig. 7:**
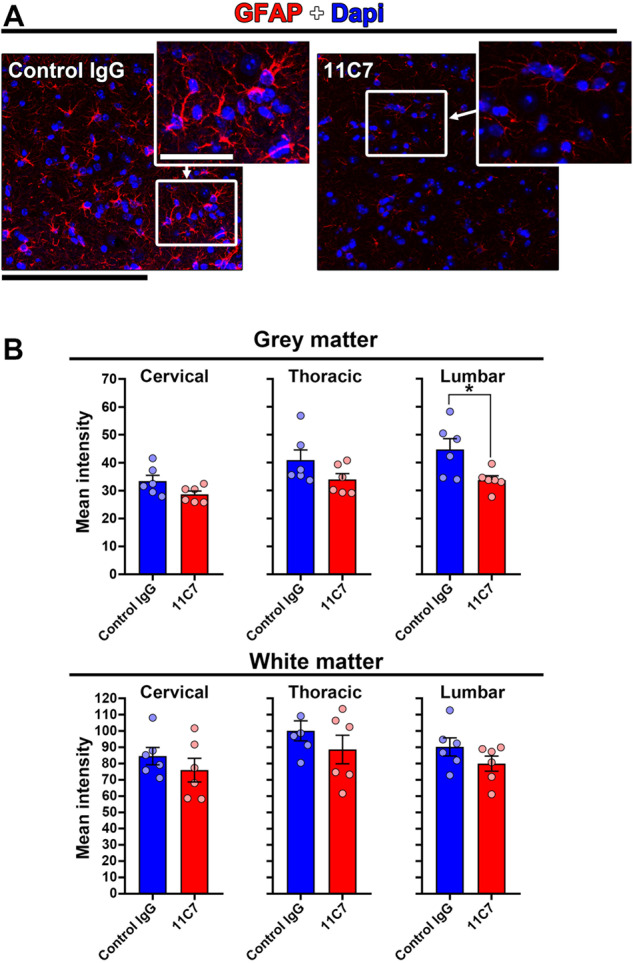
Astrocyte gliosis is reduced by 11C7. Spinal cord sections were stained for GFAP to observe astrogliosis in EAE mice treated with 11C7 mAb or control IgG. **A** Representative images of GFAP stainings showing weaker gliosis in a 11C7-treated mouse. **B** Quantitatively, the immunofluorescent signal of GFAP was lower in the lumbar spinal cord after the administration of 11C7 than control IgG antibody (unpaired *t*-test, *P < 0.05). Scale bar: 200 μm.

At the histological level, demyelination was studied on transverse spinal cord sections stained with LFB-PAS (Fig. 5A). The loss of myelin was attenuated in 11C7-treated compared with control spinal cords (Fig. 5A). This difference was particularly obvious in the lumbar region of the spinal cord where demyelination was most pronounced in control mice. These animals exhibited a gradient of demyelination between caudal-to-rostral spinal cord. Quantitatively, in the lumbar spinal cord, demyelination was reduced by more than 3-fold with 11C7 in comparison to control IgG (Fig. 5B). In control spinal cords, lumbar demyelination correlated with EAE clinical scores, suggesting that functional deficits are associated with myelin loss at this site (Fig. 5C). The effect of 11C7 on myelin preservation was confirmed by staining spinal cord sections for MBP, a protein specifically expressed in mature oligodendrocytes and abundantly present in the myelin sheath (Fig. 6A, B). In the same tissue sections, the density of motoneurons expressing β3 Tubulin (B3Tub) in the ventral gray matter was not different between control and 11C7 antibody treatments, indicating that 11C7 does not reduce EAE clinical scores by protecting motoneurons. Moreover, the infiltration of CD3^+^ T cells and Mac3^+^ monocytes did not vary between treatments (Fig. 6D–I). Interestingly, the weaker expression of Glial fibrillary acidic protein (GFAP) suggests a reduction in astrogliosis in the lumbar spinal cord of 11C7-treated EAE mice (Fig. 7). In general, our histological observations suggest that repeated intranasal 11C7 administration improves the clinical symptoms of EAE by preserving myelin and by reducing astrogliosis in the lumbar spinal cord where the lesions are normally the most severe.

### 11C7 reduces the level of Nogo-A and downstream phosphorylation of Cofilin in the cerebellum

**Fig. 8:**
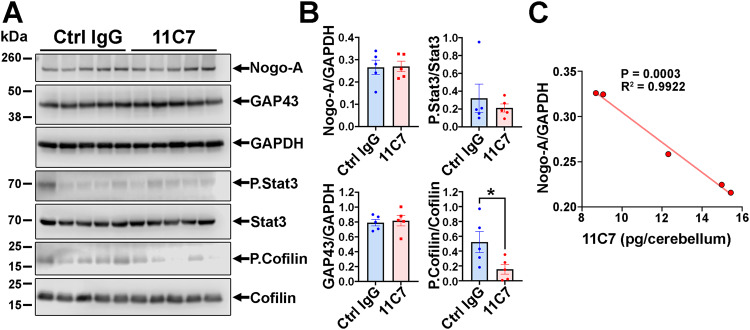
Intranasal delivery of 11C7 modifies Nogo-A signaling. **A, B** Western blot analyses were carried out for cerebellum homogenates after EAE and 30 days of antibody administration. Quantitatively, only the level of phosphorylated Cofilin (P.Cofilin/Cofilin) was significantly decreased by 11C7 (unpaired *t*-test, *P* < 0.05). **C** However, the level of Nogo-A was negatively correlated with the concentration of 11C7, assayed by ELISA.

The expression of Nogo-A, GAP43, P.Stat3/Stat3 and P.Cofilin/Cofilin were followed by Western Blotting in the cerebella of EAE mice (Fig. 8). As the cerebellum is not demyelinated (data not shown) in our EAE model, this allows to observe possible 11C7-induced downregulation of Nogo-A [25] and changes in its intracellular signaling effector P.Cofilin that is involved in F-actin remodeling [26]. GAP43 and P.Stat3/Stat3 were used as neuronal growth and inflammation signaling indicators. Our results did not show a difference in Nogo-A expression between 11C7 and control IgG-treated mice (Fig. 8A, B). However, 11C7 concentration was tightly correlated with weaker levels of Nogo-A (Fig. 8C). In addition, P.Cofilin was significantly downregulated by 11C7. The levels of GAP43 and P.Stat3/Stat3 did not differ between the two antibody treatments. The effects of 11C7 on Nogo-A and P.Cofilin are consistent with changes previously reported in neuronal cells exposed to 11C7 in vivo [25] and to Nogo-A peptides in vitro [26]. We conclude that repeated intranasal administrations of 11C7 influence the level of Nogo-A and the activation of Cofilin in the CNS of EAE mice thus, corroborating functional activity of intranasally delivered antibody.

### Intranasal IgG administration modifies brain gene expression in mouse EAE

Although demyelination is a major hallmark of EAE in the spinal cord of mice, cognitive deficits have been reported as well, presumably as a result of frontal cortex and hippocampal damage [27] and neurotransmission impairments in the brain [28]. To study the possible effects that intranasal 11C7 administration may exert on gene expression in diseased mouse brains, independently of spinal cord demyelination and motor deficits, we performed RNA sequencing (RNAseq) analyses (Fig. S1) and RT-qPCR (Fig. S2) in EAE score-matched mice treated with 11C7, control IgG or PBS. RNAseq data showed that the expression of 395 genes was modified by control IgG treatment compared with vehicle administration in the brain (Fig. S1A). This demonstrates that the delivery of control IgG can influence the expression of genes in the CNS. In addition, 11C7 specifically changed the expression level of 11 transcripts in whole brain samples (Fig. S1A, C). Thus, 11C7 caused the downregulation of *Janus kinase and microtubule interacting protein 1* (*Jakmip1*) mRNA and the upregulation of *Zinc finger protein 189* (*Zfp189*) mRNA that have been involved in GABAergic neurotransmission [29], microtubule transport [30] and cAMP-response element-binding protein (CREB) signaling [31]. Some of these changes may be due to Nogo-A signaling inhibition [32]. As the experimental groups were designed with similar EAE scores, 11C7 did not show any influence on the activation of spinal cord genes (Fig. S1B). Consistently, RT-qPCR measurements for genes susceptible to be influenced by 11C7, did not reveal significant changes in the spinal cord of EAE mice (Fig. S2). Together, these results suggest that 11C7 modifies gene expression in EAE brains, independently of the spinal cord phenotype.

## Discussion

In contrast to previous studies, where large volumes of antibodies were applied in the whole nasal cavity [16, 22], in the present study we used a more specific delivery mode by targeting the olfactory mucosa with relatively small volumes of antibody, and we followed antibody distribution in disease conditions. We observed that 11C7 administration onto the olfactory mucosa significantly reduces EAE-induced spinal cord motor symptoms and demyelination. These effects are attributable to the rapid and widespread distribution of 11C7 and its efficient inhibition of Nogo-A in the mouse brain and spinal cord. In addition, independently of the EAE spinal cord phenotype, 11C7 changed the expression of brain genes, the effect of which may influence neurotransmission. These data provide a *proof-of-concept* for the treatment of demyelinating diseases with therapeutic antibodies targeting CNS antigens through a selective intranasal pathway.

### CNS distribution of IgG after intranasal delivery

We found high levels of 11C7 in the olfactory bulb, the cerebellum and in the lumbar spinal cord after application onto the olfactory mucosa. At the olfactory epithelium, IgG transcytosis is partially mediated by the neonatal receptor of the Fc fragment (FcRn) [17, 33–35], as shown by the higher uptake observed for full-length 11C7 than its single chain variable fragment (scFv), deprived of Fc domain [17]. However, mechanisms independent of Fc-FcRn interactions may contribute to IgG transport as well [20]. The kinetic of 11C7 distribution that we observed in the CNS suggests that fast transport mechanisms operate following antibody deposition at the surface of the olfactory epithelium. Surprisingly, the CSF did not contain detectable levels of 11C7. Independently of the CSF, 11C7 may travel to the CNS regions via specific anatomical pathways such as the olfactory and trigeminal tracts [16]. The IgG distribution reported by others suggest a role of the perivascular drainage system in the rapid transport and diffusion of antibodies [16]. Whether EAE affects the transport of IgG deserves to be addressed in future experiments. In addition, antibodies recognizing CNS antigens such as 11C7 are readily retained in the nervous tissues compared with control IgG [17]. Given the accumulation of 11C7 that we observed in specific CNS areas, such as the olfactory bulb, the cerebellum and the spinal cord, we speculate that the distribution of 11C7 predominantly results from directed transport mechanisms, and to a lesser extent from its retention by Nogo-A-expressing cells (oligodendrocytes, neurons, and astrocytes).

### The influence of the intranasal route of administration on the effects of 11C7 treatment

We found that intranasal 11C7 did not influence the incidence and the onset of the disease but significantly reduced EAE clinical scores during the chronic disease phase, 25–29 days post-MOG injection. This is in contrast with the intravenous administration of anti-Nogo-A-IgG that has previously been shown to delay the onset of EAE but whose effects disappeared 24 days following MOG injection [10]. In this case, the rise in EAE symptoms may be attributable to the interruption of antibody supply and its elimination from the brain. In comparison, sustained inactivation of Nogo-A by active immunization or gene ablation afforded long-term protection [10]. In the study of Karnezis [10], the injection of large IgG bolus (400 μg/injection) had rapid therapeutic effects in contrast to intranasal delivery that was carried out with smaller amount of antibody (60 μg/injection) in our study. Although a very small fraction of IgG crosses the BBB after intravenous injection [13], the breakdown of the BBB at the EAE onset may enhance the passage of IgG from the systemic circulation to the CNS parenchyma [36], consistent with what is observed in multiple sclerosis patients [37–39]. Similarly, the BBB disruption seems to be required for the penetration of Nogo-A-targeting siRNA in the parenchyma of EAE mice [8]. If the BBB destabilization is needed for IgG and siRNA diffusion into the CNS, it may dramatically limit the duration and effectiveness of intravenously-administered reagents antagonizing Nogo-A in multiple sclerosis, as a sustained delivery seems to be required to keep EAE clinical scores at low levels [10]. It is tempting to propose that a combined strategy using both intravenous and intranasal therapeutic delivery routes may be optimal for rapid and sustained therapeutic effects in the acute and chronic phases respectively.

### Possible mechanisms underlying the effects of anti-Nogo-A antibodies on EAE

The effect of intranasal 11C7 administration in chronic EAE is of particular interest for the development of new therapies for multiple sclerosis and CNS injuries such as stroke [22]. Indeed, the stabilization of neurological deficits in the chronic phase of EAE is due to permanent demyelination and axonal damage. Currently, there is no treatment available to promote myelin and axon repair in multiple sclerosis. In our study, the reduction of EAE clinical scores was associated with a lower degree of demyelination in the spinal cord of 11C7-treated mice, consistent with other studies [8–10]. The preservation of myelinated axons in the white matter of the lumbar spinal cord is likely to enhance neuronal conductance, and hence contribute to motor function recovery in the hindlimbs and the tail. Consistent with our results, intrathecal infusion of 11C7 has been shown to activate myelin repair after lysolecithin-induced demyelination in rats [9]. In adult EAE mice, 11C7 may block the repulsive effects of Nogo-A that restricts oligodendrocyte remyelination in lesion areas, similarly to what has been described during development [40]. The processes of remyelination activated by 11C7 may operate independently of oligodendrocyte progenitor cell proliferation, migration and differentiation [9]. In the present study, the fact that anti-Nogo-A antibody treatment did not influence EAE clinical scores in the acute phase and the incidence of EAE suggests that intranasal Nogo-A may have limited effects on the activation of the immune response. The detrimental effects of Nogo-A in EAE-induced demyelination may be mediated by the activation of its receptors such as sphingosine 1-phosphate receptor 2 (S1PR2) [41]. Pharmacological or genetic inactivation of S1PR2 has been shown to promote axonal remyelination in EAE mice [11]. Downstream of S1PR2 and NgR1, the two main Nogo-A receptors, RhoA signaling leads to the activation of the F-actin depolymerizing enzyme Cofilin in neurons. Interestingly, we found a reduction in P.Cofilin in the cerebellum of mice treated with 11C7. Cofilin dephosphorylation increases its enzymatic activity and may enhance actin cytoskeleton dynamic, thus allowing neuronal plasticity and remodeling [4]. Supporting this possibility, a recent study showed that intranasal delivery of 11C7 enhanced growth and compensatory sprouting of corticofugal axon projections and functional recovery in rats with cortical stroke [22]. It would be relevant to confirm whether intranasal 11C7 is able to induce myelin and neuronal repair in a S1PR2-dependent manner via the RhoA/Cofilin pathway.

Neuronal plasticity activation and axonal regeneration may also be involved in functional recovery. Previous studies showed that Nogo-A-blocking antibody injections promoted neuronal plasticity in the spinal cord of EAE rodents [8, 9]. In a targeted model of EAE lesion in the dorsal funiculus of the cervical spinal cord, corticospinal axonal sprouting has been associated with fine motor function improvement [9]. In addition, Nogo-A has been implicated in EAE-induced axonal degeneration via Nogo66 receptor 1 activation and the intracellular phosphorylation of collapsing response mediator protein 2 (CRMP-2), a tubulin-associated protein controlling axonal growth [42]. By acting on axonal degeneration and neuronal plasticity mechanisms, 11C7 may enhance neurotransmission in EAE mice.

### Transcriptional effects of 11C7 in EAE brains

Intranasal 11C7 treatment changed the expression of a restricted number of genes in the brain. Among the 11 genes whose transcription was modified by 11C7 in the brain, we found that *Zfp189* mRNA was upregulated whereas *Jakmip1* mRNA was significantly downregulated. These genes may play a role in neurotransmission. *Zfp189* has been involved in resilience in the brain of mice subjected to social defeat [31]. Its transcription is modulated by cAMP Response Element-Binding Protein (CREB), the expression of which can reproduce the pro-resilient effects of Zfp189 upregulation in this paradigm [31]. CREB/Zfp189 signaling may be inhibited by Nogo-A in EAE. A study showed that the addition of bioactive Nogo-A fragment (delta 20) to dorsal root ganglion cells inhibited CREB phosphorylation/activation and blocked neurite outgrowth in vitro [32]. The upregulation of *Zfp189* may therefore result from 11C7-induced CREB disinhibition. This mechanism could be relevant to explore in the context of depression that affects ~50% of multiple sclerosis patients [43]. Moreover, the role of *Jakmip1* downregulation deserves to be clarified. Its implication in GABAergic neurotransmission [30] may contribute to neuronal recovery in the brain of EAE mice treated with 11C7 [44]. Independently of these genes, the higher level of *Lyn* mRNA suggests that the immune response may be modulated by 11C7 [45]. Whereas EAE brains lack high numbers of inflammatory cells, high prevalence of inflammatory spinal lesions may have interfered with the detection of specific gene changes induced by 11C7 in selected cell types.

As yet, phase III trials on remyelinating or neuroprotective approaches have failed in MS. In the case of large molecules, this may at least partly be associated with limited ability to reach the target sites in the CNS as is also known in other indications. The specific, olfactory mucosa-targeted administration of Nogo-A-neutralizing IgG directed at the olfactory mucosa improves the clinical symptoms of EAE. This non-invasive route of delivery may offer the possibility to maintain the therapeutic effects of Nogo-A-targeting antibody in multiple sclerosis.

## Materials and methods

### Production of 11C7 and control P3X antibodies

Hybridoma cells secreting 11C7, a mouse IgG1 binding and blocking the delta 20 domain of Nogo-A, was kindly provided by Prof. Martin E. Schwab (Univ. Zurich, Switzerland). Hybridoma cells were cultured in serum-free TurboDoma TP-6 medium (Cell Culture Technologies LLC, Gravesano, Switzerland) supplemented with 0.1% (v/v) Pluronic F-68 (Merck, Darmstadt, Germany) and 4 mM L-Glutamine (Lonza, Basel, Switzerland), under agitation at 37 °C in a humidified atmosphere with 5% CO_2_. Batch productions were processed with an initial seeding density of 4 ×10^5^ cells/mL. A non-binding isotype control mouse IgG1 was produced from the hybridoma cell line P3X63Ag8 (ATCC, Manassas, USA). Prior to IgG1 purification, the cell culture broth was cleared by centrifugation at 3000 × *g* for 15 min and subsequent microfiltration using a Sartopore® 2 capsule (Sartorius, Göttingen, Germany) with a heterogeneous PES double layer. The purification was performed using a Protein A MabSelect SuRe™ resin (GE Healthcare, Solingen, Germany) packed into a XK 16/40 column (GE Healthcare, Solingen, Germany) and an ÄKTA Purifier system (GE Healthcare, Solingen, Germany). After clearing, the culture broth was loaded on a resin pre-equilibrated with 10 mM phosphate buffer and 140 mM NaCl, pH 7.0. Unbound material was washed from the resin with equilibration buffer. Antibody was eluted using 20 mM sodium acetate buffer, pH 3.0. Subsequently, elution fractions were pooled and introduced to diafiltration via tangential flow filtration using a Sartocon® slice 200 stainless steel holder (Sartorius, Göttingen, Germany), equipped with a 30 kDa molecular weight cut-off Hydrosart® ultrafiltration cassette (Sartorius, Göttingen, Germany). Diafiltration of both antibody solutions was performed against phosphate buffered saline (PBS) at pH 6.5 until a final concentration of 10 mg/mL. Continuous determination of antibody concentrations was carried out with a NanoDrop™ spectrophotometer (Thermo Fisher Scientific, Dreieich, Germany) at 280 nm and applying the mass extinction coefficient 13.7 L/(g • cm). Both antibodies were formulated in PBS at pH 6.5 and at a concentration of 10 mg/mL for intranasal administration.

### Animals

Adult C57BL/6J female mice were purchased from Janvier (France) and were used at 2 months of age. Animal experiments were approved by the local authorities (Veterinary Office of the Canton of Bern, Switzerland, licence #BE88/19).

### Experimental autoimmune encephalomyelitis (EAE) induction

To induce EAE, mice were subcutaneously injected with 100 μg of recombinant myelin oligodendrocyte glycoprotein peptide (MOG_35-55_, Institute of Medical Immunology, Charité Berlin, Germany) in a solution containing complete Freund’s adjuvants (CFA), including 100 µg of *Mycobacterium tuberculosis* (H37RA, Difco, Detroit, Michigan, USA). On the same day and two days later, 300 ng of pertussis toxin were intraperitoneally injected (Quadratech, Epsom, United Kingdom). EAE clinical symptoms were assessed every day using the following scoring method: 0 = no visual sign of disease, 0.5 = tail paralysis, 1 = paraparesis, 2 = hindlimb paralysis, 3 = hindlimb paralysis and incontinence. Statistical differences in EAE clinical scores were determined between control IgG and 11C7 in the GraphPad Prism software, using a two-way ANOVA and Bonferroni’s multiple comparison test.

### Intranasal administration of IgG

Intranasal administrations of IgG were carried out using a refined method allowing to target the olfactory region, as recently described [17, 46]. In brief, IgGs were delivered on the olfactory mucosa using a pediatric catheter (Nutriline Twinflow, Vygon GmbH und Co KG, Aachen, Germany) adapted to a 10-μL Hamilton syringe (VWR International GmbH, Darmstadt, Germany), under isoflurane anesthesia lasting ~2 min. Animals received daily administrations of 30 μg of control IgG or 11C7 [6] antibodies diluted in 3 μL of PBS in each nostril (60 μg/day/animal). To avoid nasal irritation, a balm (Bepanthen Augen- und Nasensalbe, Bayer Vital GmbH, Germany) was applied at the tip of the catheter. The catheter was inserted with an angle of 20–25° at ~8 mm into the nostril. Fifteen seconds after application, the catheter was slowly withdrawn. EAE experiments were repeated 3 times with groups of 7–10 mice for each treatment. Similar animal numbers were sufficient in previous studies to observe differences after intranasal administrations of anti-Nogo-A vs isotype control IgG [22] and after EAE induction [8, 10]. Prior to EAE induction, groups were constituted of mice with similar body weight. No animal was excluded from statistical analyses. Experiments were carried out in blind.

### Histology

At the end of the experiment, 30 days after MOG immunization, mice received an overdose of pentobarbital before intracardial perfusion with PBS and 4% paraformaldehyde. The spinal cord was rapidly dissected and postfixed overnight. After tissue embedding in paraffin, 5-μm thick transverse sections were cut, in cervical, thoracic and lumbar spinal cord regions and stained with Luxol Fast Blue (LFB) and counterstained with Periodic Acid Schiff (PAS). On adjacent sections, immunofluorescent stainings were carried out by applying a rabbit anti-myelin binding protein (MBP) antibody (abcam, cat# ab218011, 1:5000) and a mouse anti-β3 tubulin (B3Tub) antibody (Promega, cat#G712A, 1: 1000). After PBS washes, spinal cord slices were incubated with appropriate secondary antibodies. Images were acquired with a slide scanner (Pannoramic 250 Flash III, 3DHISTECH, Budapest, Hungary). The percentage of demyelination was determined in blind by measuring the surface of LFB-free tissue in the white matter with the CaseViewer 2.4 (3DHISTECH) software. The number of motoneurons expressing B3Tub was counted in the gray matter of the ventral horn. Three sections per segment were used for quantitative analyses. Statistical significance was determined using unpaired *t-tests* in GraphPad Prism software.

### Mouse brain clearing

Brains were incubated in a hydrogel solution containing 10% acrylamide (vol/vol), 2.5% bis-acrylamide (vol/vol) and 0.25% VA044 (wt/vol) in PBS, at 4 °C for 3 days. Oxygen was removed in brains and replaced with nitrogen. Hydrogel polymerization was induced at 37 °C for 3 h. Embedded brains were then placed in a clearing solution containing 4.4% (wt/vol) sodium dodecyl sulfate (SDS) and 1.2% (wt/vol) boric acid in ultra-pure water, pH 8.5, at 37 °C . Specimens were gently shaken during the whole clearing procedure, for 3–4 weeks. When the samples appeared sufficiently transparent, they were incubated for 1 day in PBS with 0.1% Triton-X (PBST) and for 1 day in PBS, to remove SDS. Finally, mouse brains were optically cleared with sequential immersions in solutions containing 20% and 40% 2-2′ Thiodiethanol (TDE) in PBS, for 1 day under agitation. The 40% TDE solution was used as index-matching solution for imaging [47].

### Whole-mounted brain immunofluorescence

Before homogenizing the refractive index of each sample, whole-mounted brains were immunolabelled. Brains were first washed in PBST at room temperature (RT) overnight. Then, cleared samples were incubated with an anti-mouse antibody coupled to Alexa647 (Thermofisher, 1:1000) in PBST at 37 °C for 1 week. This last step was repeated twice. Brains were extensively washed in large volume of PBST at RT for one day. Before imaging with the light-sheet microscope, the specimens were optically cleared with a solution containing 40% TDE in PBS.

### Light-sheet microscopy

The custom-made light-sheet microscope used in the experiments has been described in detail by Mullenbroich and colleagues [48]. Briefly, the sample was illuminated from the side using a virtual light sheet created with a galvo scanner (6220H, Cambridge Technology, Bedford, MA), which was coupled via a 4f system to an air objective (Plan Fluor EPI 10X NA 0.3, Nikon) covered with a protective coverslip. Light emitted from the specimen was detected orthogonally to the illumination plane using an immersion objective corrected for clearing solutions (XLPLN10XSVMP 10X NA 0.6, Olympus, Tokyo, Japan). Then, it was bandpass-filtered to isolate fluorescence light and projected by a tube lens onto the chip of a sCMOS camera (Orca Flash 2.0, Hamamatsu) operating in rolling-shutter mode to guarantee confocal line detection. During imaging, the sample was fixed in a refractive-index-matched quartz cuvette (3/Q/15/TW, Starna Scientific, Hainault, United Kingdom) and moved using a set of high-accuracy linear translators (M-122.2DD, Physik Instrumente, Karlsruhe, Germany). Defocus correction was implemented using RAPID. The entire system was controlled by custom software written in LabVIEW 2012 using the Murmex library (Distrio, Amsterdam, The Netherlands). The software can be freely downloaded from https://github.com/ludovicosilvestri/RAPID_CLSM. Tiled images acquired with the microscope were stitched together using ZetaStitcher (https://github.com/lens-biophotonics/ZetaStitcher).

### Image analysis

Acquired images were analyzed using Fiji (ImageJ) software. Sagittal stacks were re-sliced from the top and two different thresholds were set to discriminate the vasculature (high fluorescence intensity values) and the neuropil (low intensity values). The ZROIaxis profiler plug-in was then used to measure the mean fluorescence intensity per slice across the differently thresholded images. The mean intensity fluorescence was then calculated for 50 slices windows in the rostral, medial and caudal part of each brain, and values were normalized with respect to the highest average intensity recorded in each set of experiments including brains from mice treated under different conditions.

### Capture ELISA for 11C7

A capture ELISA was used to determine the concentration of 11C7 in the plasma and CNS tissues of intact mice receiving intranasal applications of 11C7 (60 µg/day) or control IgG for 5 consecutive days. The sensitivity was 0.06 ng/mL. After the injection of an overdose of pentobarbital, blood and CNS tissues were collected. Blood was obtained by intracardial puncture. Then, mice were perfused with PBS to remove blood before dissection of CNS tissues and snapfrozen in liquid nitrogen. Brain and spinal cord lysates were prepared in CHAPS buffer (20 mM Tris-HCl, 0.5% CHAPS, pH 8.0). Plasma was collected in fresh tubes after blood centrifugation at 4000 × *g*, 4 °C. For 11C7 concentration assay by capture ELISA, 96-well plates (M9410, Sigma-Aldrich) were coated overnight at 4 °C with human delta 20 fragment (MAHHHHHHLVPRGSAVANMPEGLTPDLVQEACESELNEVTGTKIAYETKMDLVQTSEVMQESLYPAAQLCPSFEESEATPSPVLPDIVMEAPLNSAVPSAGASVIQPSSSPLEASSVNYESIKHEPENPPPYEEAMSVSLKKVSGIKEEIKEPENINAALQETEAPYISIACDLIKETKLSAEPAPDFSDYSEMAKVEQPVPDHSELVEDSSPDSEPVDLFSDDSIPDVPQKQDETVMLVKESLTETSFESMIEYENKEKLSALPPEGGKPYLESFKLSLDNTKDTLLPDEVSTLSKKEKIPLQMEELSTAVYSNDDLFISKEAQIRETETFSDSSPIEAGLNDIFEAQKIEWHEHHHHHH) or rat Nogo-A aa 623-640 fragment (SYDSIKLEPENPPPYEEA) against which 11C7 has been raised [12]. Wells were washed with PBS, pH 7.4, and 0.01% of Tween20. Unspecific binding was blocked with 5% skimmed milk in Tris Buffered Saline (TBS), pH 7.4. After 2 washes, 100 µL of diluted samples were applied in each well and incubated for 2 h at room temperature under gentle agitation. After 3 washes, goat anti-mouse Fc antibody conjugated to horseradish peroxidase (HRP) (AP127P, Merck Millipore, 1: 5000) was added in TBS containing 5% skimmed milk. After extensive washes, 3,3′,5,5′-tetramethylbenzidine (N301, Thermofisher Scientific) was added as HRP enzymatic substrate. The enzymatic reaction was stopped after 30 min of incubation with 2 N/1 M sulfuric acid. Absorbance was measured at 450 nm with a plate reader (SpectraMax M5e, Molecular Devices). Standard curves were established using known concentrations of 11C7.

### RT-qPCR

Under deep anesthesia with pentobarbital, animals were intracardially perfused with PBS and brain or spinal cord tissues were rapidly dissected, placed in plastic tubes and flash-frozen in liquid nitrogen. Tubes were stored at −80 °C until RNA extraction. Total RNA was obtained from the right hemisphere of the brains and from the lower part of the spinal cords by using a miRNeasy micro isolation kit (Qiagen, Basel, Switzerland). Residual genomic DNA was eliminated by DNase treatment (Qiagen). Oligo (dt) and M-MLV reverse transcriptase (Fisher Scientific, Basel, Switzerland) were used to transform equal amounts of RNA for reverse transcription. Amplification of ten nanograms of cDNA with the SYBR Green I Master polymerase ready mix (Roche Diagnostics AG, Rotkreuz, Switzerland) was done using the Light Cycler 480 thermocycler (Roche Diagnostics AG). Primer pairs were designed to span the intronic sequences or to cover exon-intron boundaries (see Supplementary Table S2 for sequences). The comparative threshold cycle (ΔΔCT) method was used to calculate the relative quantification. As a reference gene and control sample, *Gapdh* was used to normalize cDNA levels. Each reaction was done in triplicate for 4 mice per condition. Statistical analysis was performed using a one-way ANOVA followed by Tukey post hoc test (GraphPad Prism).

### Transcriptomic analysis (total RNA sequencing)

The quantity and quality of the extracted RNA was assessed using a Thermo Fisher Scientific Qubit 4.0 fluorometer with the Qubit RNA BR Assay Kit (Thermo Fisher Scientific, #Q10211) and an Advanced Analytical Fragment Analyzer System using a Fragment Analyzer RNA Kit (Agilent Technologies AG, #DNF-471, Basel, Switzerland), respectively. Sequencing libraries were produced using an illumina TruSeq Stranded mRNA Library Prep kit (Illumina, #20020595, Zürich, Switzerland) in combination with TruSeq RNA UD Indexes (Illumina, #20022371) according to Illumina’s guidelines. Pooled cDNA libraries were sequenced paired-end using an Illumina NovaSeq 6000 S2 Reagent Kit (300 cycles; Illumina, #20028314) on an Illumina NovaSeq 6000 instrument, generating an average of 125 million reads/sample. The quality of the sequencing run was assessed using Illumina Sequencing Analysis Viewer (Illumina version 2.4.7) and all base call files were demultiplexed and converted into FASTQ files using Illumina bcl2fastq conversion software v2.20. The quality control assessments, generation of libraries and sequencing run was performed at the Next Generation Sequencing Platform, University of Bern. Quality of sequencing reads was assessed with fastqc (0.11.9). Raw sequencing reads were aligned to the mouse reference genome version GRCm38 (Ensembl) with hisat2 (2.1.0) while setting the strandedness option to ‘RF’. Fragment counting of read alignments in gene regions (annotation release 101) was performed with featureCounts (1.6.0), while setting strandedness to 2 (synonym to ‘RF’) and minimum mapping quality to 10. Further downstream analyses and visualization were performed with R (4.1.0) [49]. The count matrix was used for differential gene expression analysis in both tissues (brain and spinal cord) separately with DESeq2 (1.30.1) [50].

### Western blot analysis

The cerebella were isolated after intracardiac perfusion with PBS, weighed and lysed in CHAPS buffer with anti-protease/phosphatase inhibitors (0.4 mL CHAPS/ 100 mg tissue weight). After 1 h on ice, the lysates were centrifuged at 15,000 rpm for 15 min at 4 °C. The supernatants were then transferred into a clean Eppendorf and protein assay (Bio-Rad) was performed. Cerebellum proteins (20 μg/well) were separated by electrophoresis in a 4–12% Bis-Tris NuPAGE gel (ThermoFisher Scientific). After protein transfer, nitrocellulose membranes were pre-incubated in a blocking solution (Tris-base 0.1 M, 0.2% Tween 20, pH 7.4 and 5% BSA) for 1 h at room temperature. Membranes were then incubated with primary antibodies overnight at 4 °C (Bianca rabbit Nogo-A antibody, generous gift from Prof. M.E. Schwab, Zürich, Switzerland; rabbit anti-Gap43 antibody, Sigma #ab5220; mouse anti-GAPDH antibody, Abcam #ab8245; rabbit anti-P.Stat3 antibody, Cell Signaling #9131; rabbit anti-Stat3 antibody, Cell Signaling #12640; rabbit anti-P.cofilin antibody, Cell Signaling #3313; rabbit anti-cofilin antibody, Cell Signaling #5175). The day after, after washes, membranes were incubated for 1 h at room temperature with the appropriate horseradish peroxidase-conjugated secondary antibody (ThermoFisher Scientific). The LiCor Western Sure Premium Chemiluminescent Substrate (LiCor BioSciences GmbH, Bad Homburg, Germany) was used to detect chemiluminescent bands in a LiCor C-Digit blot scanner (LiCor). Band signals were quantified with the ImageJ software and analyzed with the GraphPad Prism software.

### Supplementary information

FIGURE S1

FIGURE S2

Table S1

Table S2

ull and uncropped western blots-CDDiscovery.pdf

## Data Availability

The datasets used and/or analyzed during the current study are available from the corresponding author on reasonable request.
