# Supplementary figures and images for: Nogo-A antibody delivery through the olfactory mucosa mitigates experimental autoimmune encephalomyelitis in the mouse CNS
Source: Cell Death Discov. 2023 Aug 9;9:290. doi: 10.1038/s41420-023-01588-7 (PMC10412545; doi:10.1038/s41420-023-01588-7)

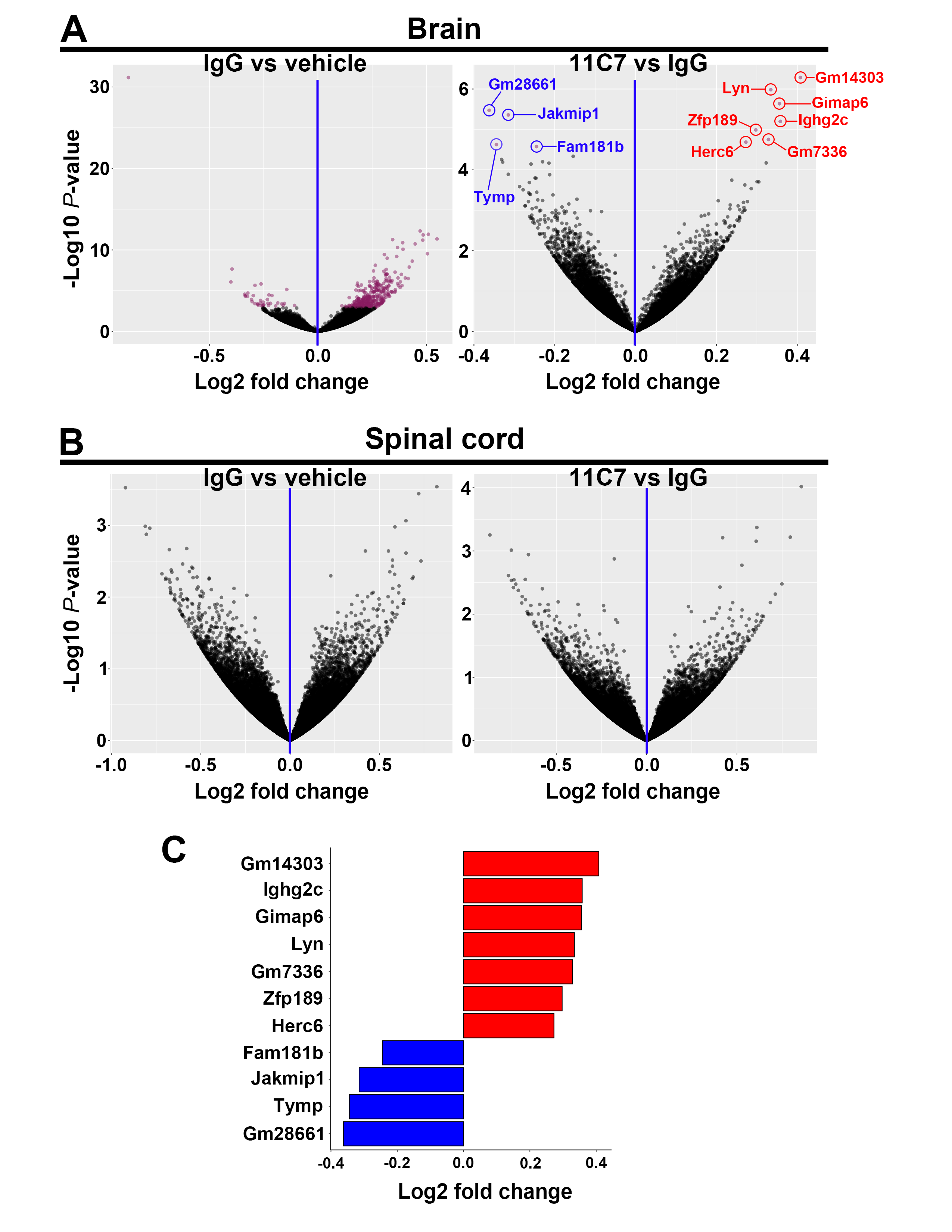

Supplement: Supplementary file 1 — FIGURE S1 [file 41420_2023_1588_MOESM1_ESM.tif]

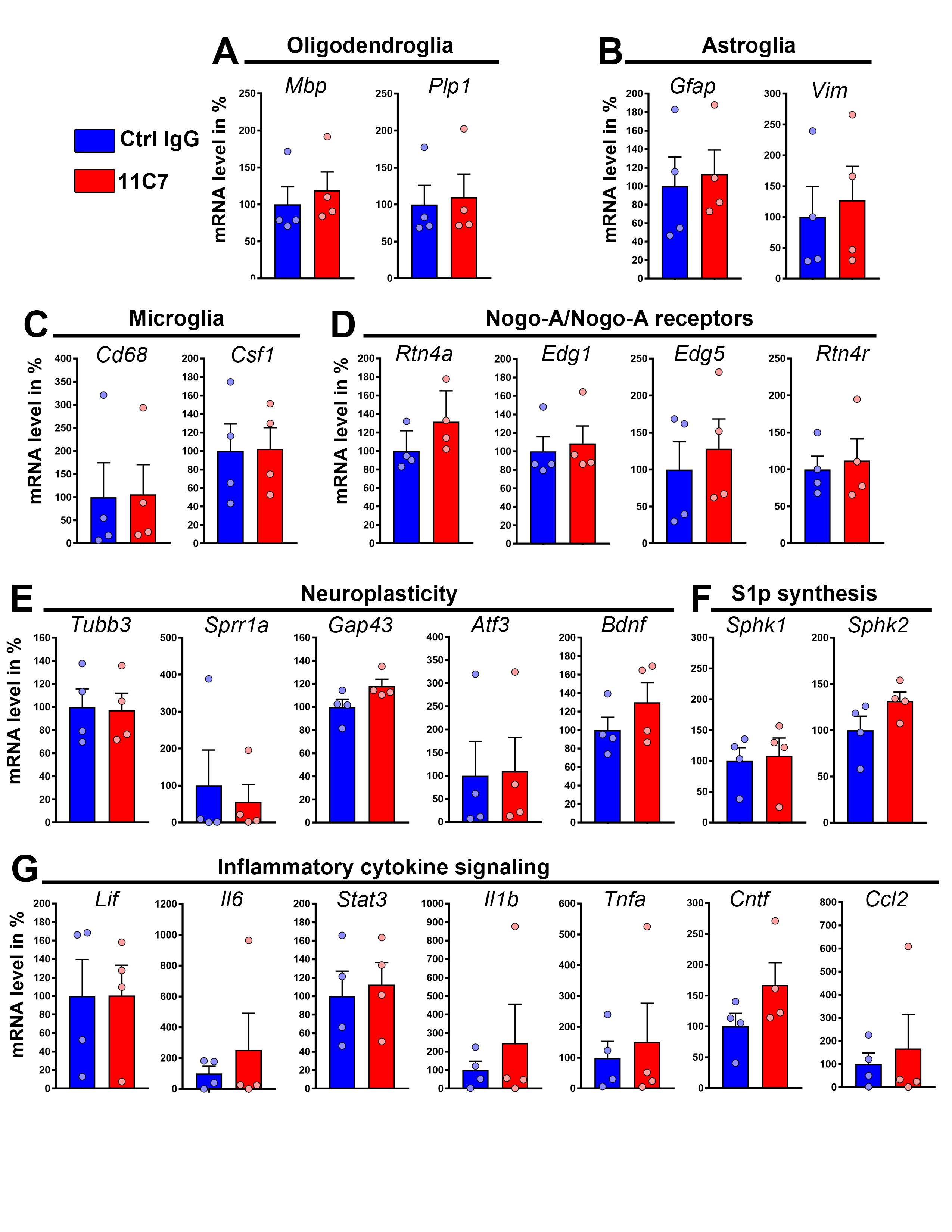

Supplement: Supplementary file 2 — FIGURE S2 [file 41420_2023_1588_MOESM2_ESM.tif]

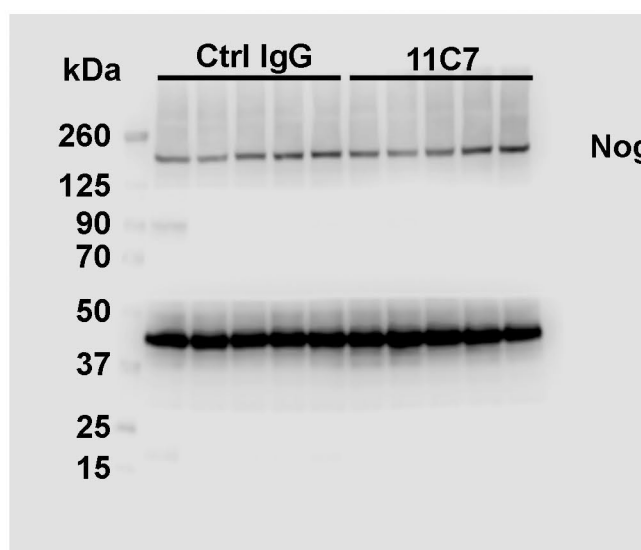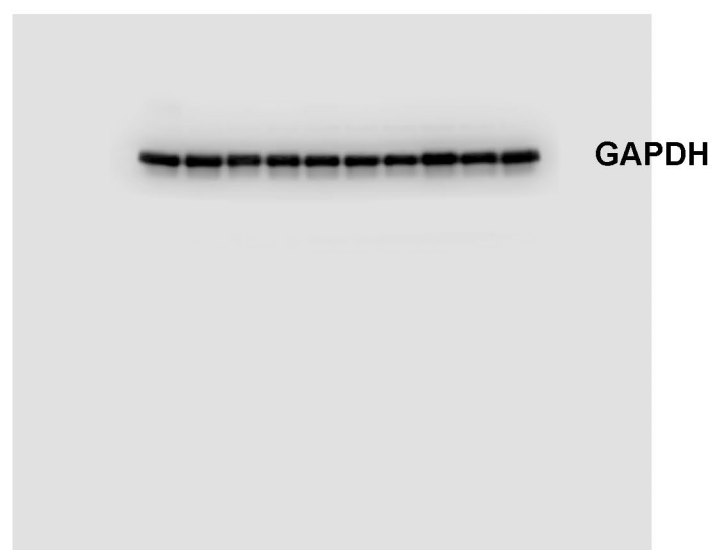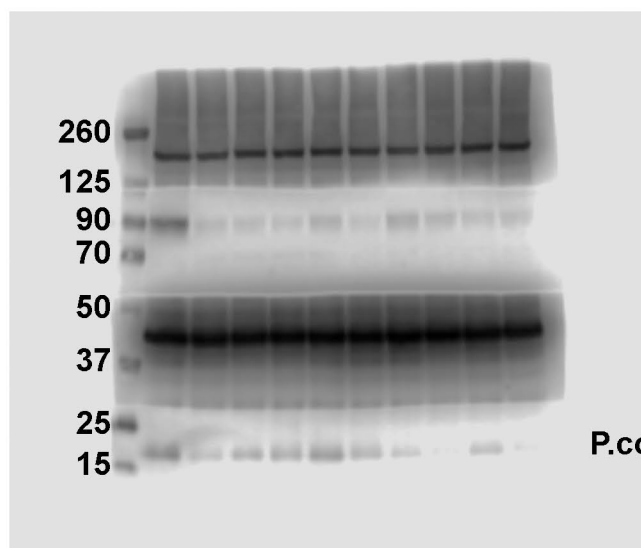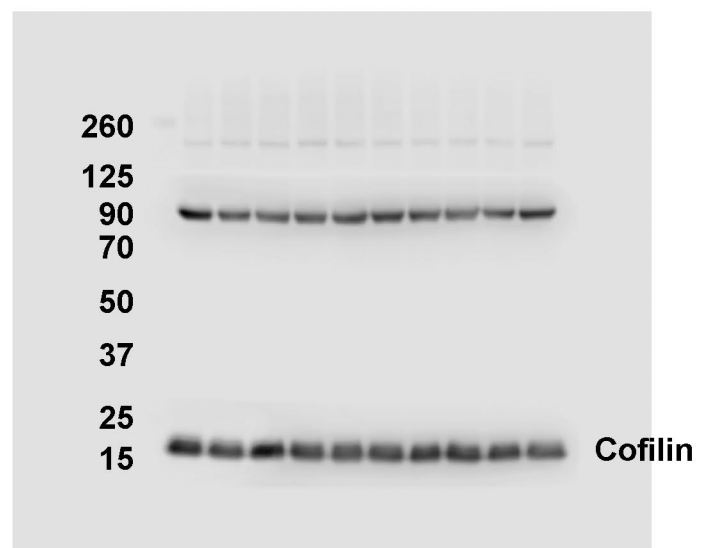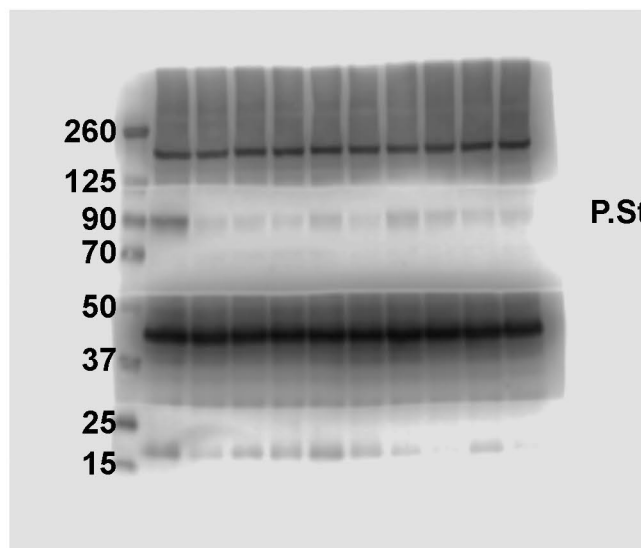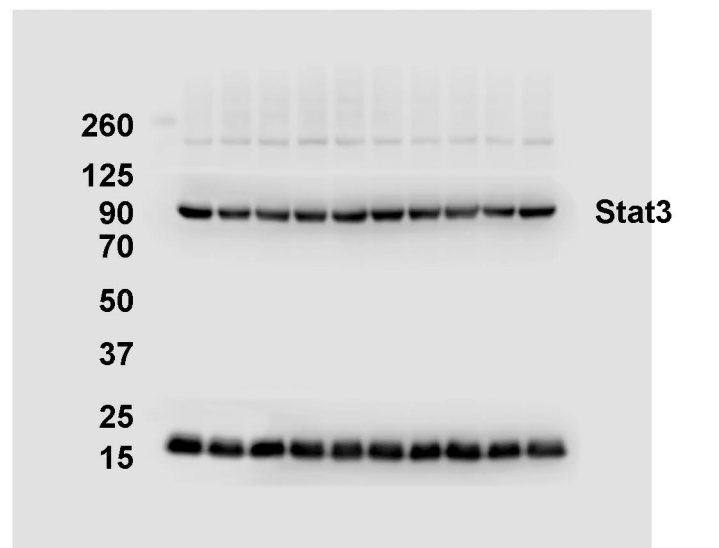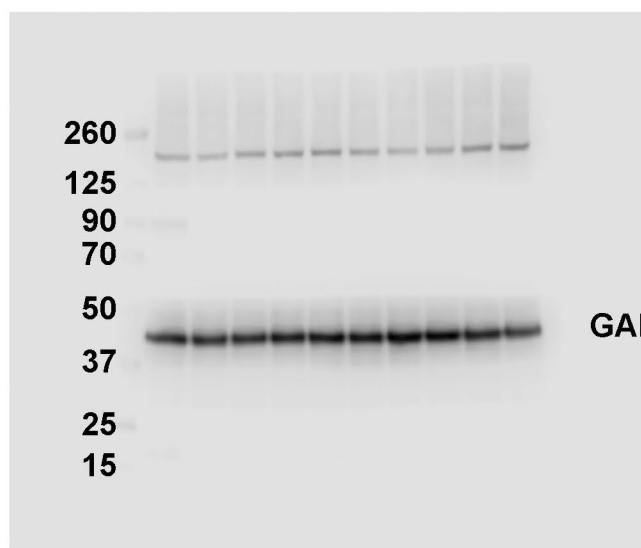

Supplement: Supplementary file 5 — ull and uncropped western blots-CDDiscovery.pdf [file 41420_2023_1588_MOESM5_ESM.pdf]
