# Supplementary material for: Nogo-A antibody delivery through the olfactory mucosa mitigates experimental autoimmune encephalomyelitis in the mouse CNS
Source: Cell Death Discov. 2023 Aug 9;9:290. doi: 10.1038/s41420-023-01588-7 (PMC10412545; doi:10.1038/s41420-023-01588-7)
Supplement: Supplementary file 3 — Table S1 [file 41420_2023_1588_MOESM3_ESM.docx]

**Supplementary table S1-ELISA measurements of total [IgG] and 11C7 mAb in the CSF and plasma 1h after administration on the olfactory mucosa.** The CSF was collected from the cisterna magna. For 11C7 ELISA, rat Nogo-A aa 623-640 fragment was used as a bait.
