# Supplementary material for: Nogo-A antibody delivery through the olfactory mucosa mitigates experimental autoimmune encephalomyelitis in the mouse CNS
Source: Cell Death Discov. 2023 Aug 9;9:290. doi: 10.1038/s41420-023-01588-7 (PMC10412545; doi:10.1038/s41420-023-01588-7)
Supplement: Supplementary file 4 — Table S2 [file 41420_2023_1588_MOESM4_ESM.docx]

| **Gene names** | **Forward primer (5’-3’)** | **Reverse primer (5’-3’)** |
| --- | --- | --- |
| *Gapdh* | cagcaatgcatcctgcacc | tggactgtggtcatgagccc |
| *Atf3* | ACCTCCTGGGTCACTGGTATTTG | TTCTTTCTCGCCGCCTCCTTTTCC |
| *Bdnf* | CAAAGCCACAATGTTCCACCAG | GATGTCGTCGTCAGACCTCTCG |
| *Ccl2* | GGCTCAGCCAGATGCAGTTA | CTGCTGCTGGTGATCCTCTT |
| *Cd68* | ACCTACATCAGAGCCCGAGTACAG | TTCTGCGCCATGAATGTCCACTG |
| *Cntf* | CTCTGTAGCCGCTCTATCTG | GGTACACCATCCACTGAGTC |
| *Csf1* | GCTCCAGGAACTCTCCAATA | TCTTGATCTTCTCCAGCAGC |
| *Edg1* | TCAGGGAACTTTGCGAGTGA | AACAGCAGCCTCGCTCAAG |
| *Edg5* | CATCGCCATCGAGAGACAAG | TCAGACAATTCCAGCCCAGG |
| *Gap43* | TGCTGTCACTGATGCTGCT | GGCTTCGTCTACAGCGTCTT |
| *Gfap* | CCACCAAACTGGCTGATGTCTAC | TTCTCTCCAAATCCACACGAGC |
| *Il6* | ACCGCTATGAAGTTCCTCTC | CTCCGACTTGTGAAGTGGTA |
| *Il1b* | GCTATGGCAACTGTTCCTGA | GATGTGCTGCTGCGAGATT |
| *Lif* | AATGCCACCTGTGCCATACG | CAACTTGGTCTTCTCTGTCCCG |
| *Mbp* | CACACACGAGAACTACCCA | GGTGTTCGAGGTGTCACAA |
| *Plp1* | tcagtctattgccttccctagc | agcattccatgggagaacac |
| *Rtn4* | cagtggatgagaccctttttg | gctgctccttcaaatccataa |
| *Rtn4r* | CTCGACCCCGAAGATGAAG | TGTAGCACACACAAGCACCAG |
| *Sphk1* | ATACTCACCGAACGGAAGAAC | ATTAGCCCATTCACCACCTC |
| *Sphk2* | GCTTTACGAGGTGCTGAATG | AGAAGCGAGCAGTTGAG |
| *Sprr1a* | GAACCTGCTCTTCTCTGAGT | AGCTGAGGAGGTACAGTG |
| *Stat3* | CAAAACCCTCAAGAGCCAAGG | TCACTCACAATGCTTCTCCGC |
| *Tnf* | CCACGCTCTTCTGTCTACTGA | GGCCATAGAACTGATGAGAGG |
| *Tubb3* | GGCCTCCTCTCACAAGTATG | TTGCCAGCACCACTCTGAC |
| *Vim* | TACAGGAAGCTGCTGGAAGG | TGGGTGTCAACCAGAGGAA |

**Supplementary table 2 – Primer pair sequences**
